# Improvement After Hardware Removal in Post-Fusion Adult AIS: A Unique 35-Year Case Study Using Schroth-Based Physiotherapy and Bracing

**DOI:** 10.3390/healthcare14010043

**Published:** 2025-12-24

**Authors:** Josée Boucher, Andrea Lebel, Dat Nhut Nguyen, Stéphanie Jacques, Jacques Charest, Sarah Shidler, Carole Chebaro, Chun Han Huang, Nadina Adulovic, Jacob Carberry

**Affiliations:** 1 Centre Intégré de Santé et Services Sociaux de l’Abitibi-Témiscamingue, Hôpital de Rouyn-Noranda, Rouyn-Noranda, QC J9X 2B2, Canada; dat.nhut.nguyen.med@ssss.gouv.qc.ca (D.N.N.); stephanie.jacques.med@ssss.gouv.qc.ca (S.J.); 2 Scoliosis Physiotherapy and Posture Centre, Ottawa, ON K1Z 8N8, Canada; 3 Faculty of Health Sciences, Université du Québec en Abitibi-Témiscaminque, Rouyn-Noranda, QC J9X 5E4, Canada; 4 Neuro Core Physiotherapy, Richmond Hill, ON L4B 0A9, Canada; carole@neurocore.ca; 5 Hugo Spine Clinic Taichung, Taichung City 404524, Taiwan; handy1229@gmail.com; 6 Faculty of Sciences, Ottawa University, Ottawa, ON K1N 6N5, Canada; nadina.adulovic@icloud.com; 7 Faculty of Medicine, Palacky University, 779 00 Olomouc, Czech Republic; jacobcarberry0@gmail.com

**Keywords:** adult scoliosis, post-fusion spinal deformity postural deterioration, Schroth method, physiotherapeutic scoliosis-specific exercises (PSSE), bracing, adult scoliosis conservative management, infection, complications post-removal of hardware, spinal fusion, sagittal imbalance

## Abstract

**Highlights:**

**What are the main findings?**

- Long-term progression occurred after hardware removal in adult scoliosis.
- Patient declined revision surgery due to elevated complication risks.
- Schroth-based physiotherapy and bracing were used over a 28-month period.

**What are the implication of the main findings?**

- Structural, respiratory, and functional outcomes significantly improved.
- Non-surgical care restored autonomy and avoided a third spinal operation.

**Abstract:**

**Background:** Adult scoliosis following spinal fusion and subsequent hardware removal presents complex challenges, especially when deformity progresses in both the sagittal and coronal planes. Revision surgery is often recommended but it entails considerable risks. Conservative approaches, including Schroth-based physiotherapy and bracing, offer promising alternatives for select patients, particularly older adults with comorbidities or those who decline further surgery. **Case Presentation:** A woman with adolescent idiopathic scoliosis (diagnosed at age 13) underwent spinal fusion with Harrington rod instrumentation and costoplasty at age 24. She was de-instrumented two years later due to infection and developed progressive thoracic hyperkyphosis, coronal imbalance, and 12 cm loss of height over two decades. At age 47, she chose conservative management instead of revision surgery. **Methods:** She completed a 28-month program combining Schroth-based physiotherapy supervised by a certified therapist and part-time bracing. Outcomes included radiographs, inclinometry, spirometry, spinal-muscle ultrasound, height/posture measures, and SRS-22 and ODI scores. **Results:** Substantial clinical improvements were observed. Thoracic kyphosis decreased from 80° to 45° (44%) and the coronal thoracic curve was reduced from 48° to 32° (33%). Lumbar lordosis increased by 6°, standing height rose by 4 cm, and forced vital capacity improved by 900 mL (33%). The Oswestry Disability Index (ODI) score improved from 4% to 0%, and SRS-22 domains (pain, function, self-image, mental health, satisfaction) showed meaningful gains. The patient returned to full physical activity and avoided further surgery. **Conclusion:** This case highlights the potential of Schroth-based physiotherapy with bracing to reverse postural and functional decline in complex post-fusion scoliosis. It offers a viable non-surgical option when revision surgery poses a high risk or is declined and aligns with modern rehabilitative goals focused on long-term quality of life.

## 1. Introduction

Adult scoliosis following spinal fusion presents significant long-term management challenges, especially after hardware removal. While posterior spinal instrumentation provides initial stabilization and correction of spinal deformities, hardware-related complications such as infection, mechanical failure, or chronic pain may necessitate implant removal [1,2,3,4,5,6,7,8,9], exposing patients to a high risk of structural destabilization; sagittal and coronal decompensation; junctional pathologies including proximal junctional kyphosis (PJK), proximal junctional flexion (PJF), and distal junctional kyphosis (DJK); and long-term disability, particularly in the absence of re-instrumentation [3,5,8,10,11,12,13].

Despite its clinical relevance, progression after hardware removal is poorly documented. Available reports show consistent radiographic deterioration and functional decline [2,3,6,8,11]. Muschik et al. [3], for example, reported worsening spinal deformities in patients who underwent hardware removal due to infection, even when re-instrumentation was attempted, highlighting the mechanical and biological vulnerability of the post-fusion spine. Moreover, revision surgery carries high complication rates, including pseudarthrosis, reduced muscle integrity, and diminished quality of life [2,14,15,16,17].

While the 2016 Scientific Society on Scoliosis Orthopaedic and Rehabilitation Treatment (SOSORT) Guidelines [18] support scoliosis-specific exercises and bracing in adolescents and advocate for a multidisciplinary, patient-centered approach, no current guidelines—from SOSORT [18], the Scoliosis Research Society (SRS) [19], or the International Society for Prosthetics and Orthotics (ISPO)—address adults with post-fusion scoliosis or post-hardware removal. This gap is notable in patients at an elevated surgical risk.

Conservative management, particularly physiotherapy scoliosis-specific exercises (PSSE), such as the Schroth method, has gained attention as a non-invasive strategy for adult spinal deformities [20,21,22,23]. Schroth emphasizes three-dimensional postural correction, axial elongation, and respiratory optimization. While several trials support its use in adolescents [24,25,26,27,28], its application in post-fusion scoliosis remains sparsely documented, with only two cases explored in non-fused adults [29,30], and a single case report has addressed its use after spinal fusion [31]. Ongoing trials in adult degenerative scoliosis (NCT03862417) exclude post-fusion and hardware removal cases. Bracing has been investigated in adults with spinal deformity [32,33,34,35,36,37], hyperkyphosis, and non-physiological lumbar conditions, but no published data exist on brace use in adult post-fusion and post-hardware removal patients. The absence of clinical trials or evidence-based guidelines targeting these subgroups underscores the significant lack of data in the current literature and highlights the relevance of documenting detailed clinical trajectories, as in the present report.

This study presents the 35-year longitudinal course of a woman with adolescent idiopathic scoliosis who developed progressive structural collapse after hardware removal. Managed exclusively with Schroth-based physiotherapy and targeted bracing, she achieved measurable improvements in spinal alignment, respiratory capacity, and quality-of-life outcomes.

This issue is increasingly relevant in an aging population, where the adult scoliosis prevalence is rising and many patients have complex histories—prior surgery, infections, and comorbidities—that increase the surgical risk [38].

The aim of this study is to describe structural, functional, and quality-of-life outcomes after a 28-month conservative program in a patient with progressive post-fusion scoliosis and hyperkyphosis following hardware removal and to highlight the potential of Schroth-based physiotherapy and bracing as alternatives to revision surgery in complex adult cases.

## 2. Case Presentation

### 2.1. Initial Diagnosis and Early Management

In 1989, a 13-year-old girl was diagnosed with adolescent idiopathic scoliosis (AIS) after her dance teacher noted a right-sided rib prominence hump, confirmed by the Adams forward bending test. At the time, routine scoliosis screening had been discontinued in Canadian schools, contributing to a late diagnosis rate of approximately 32% [39]. Initial radiographs showed 30° Cobb right thoracic and 20° Cobb left lumbar curves. She received conservative management consisting of Global Postural Re-education (GPR)-based physiotherapy and semi-annual radiographic follow-up; bracing was not recommended.

### 2.2. Curve Progression and First Surgical Intervention

Over the next decade, curves progressed to 47° Cobb thoracic and 38° Cobb lumbar. At age 24, she underwent posterior fusion from T5 to L2 with Harrington instrumentation and right costoplasty (ribs 6–9). Post-operative radiographs showed correction to 20° Cobb for both curves. Thoracic kyphosis remained at 57°, and lumbar lordosis increased from 62° to 71°, approaching her pelvic incidence of 62°; optimal sagittal harmony is maintained when LL corresponds to PI within about ±9° [40]. Standing height increased from 172 cm to 178 cm.

### 2.3. Post-Operative Complications and Hardware Removal

Two years post-fusion (2002), post-partum complications (intravaginal hematoma and endometritis) were followed by severe back pain with normal radiographs. In early 2003, she developed wound dehiscence and deep infection; imaging showed a broken screw, osteomyelitis, and partial hardware detachment. Hardware removal was performed (L2 screw fragment retained), followed by debridement and six weeks of IV antibiotics. Cultures were negative. After revision, within nine months, the curves increased from 20° to 37° thoracic and from 20° to 31° lumbar and progressed further despite GPR, reaching 48° and 34°, respectively, by 2016.

### 2.4. Progressive Postural Collapse Following Hardware Removal

Following hardware removal, she developed paravertebral spasms, neuropathic thoracolumbar and periscapular pain, intermittent nocturnal upper-limb numbness, neck pain, and progressive trunk collapse with rib–iliac contact. Between 2003 and 2022, sagittal alignment and standing height declined substantially, indicating pathological spinal collapse beyond what would normally be anticipated with aging [41,42]. Height declined from 178 cm to 166 cm, thoracic kyphosis increased from 43° to 80°, and degenerative changes became apparent by 2009.

### 2.5. Baseline Structural and Functional Status Prior to Schroth-Based Physiotherapy

Upon initiation of Schroth therapy in November 2022, she exhibited advanced postural collapse with severe sagittal imbalance. Inclinometry showed thoracic kyphosis of 80°, a 42° increase since 2003; no post-removal inclinometric data were available following the revision, which limited post-removal comparison.

**Figure 1:**
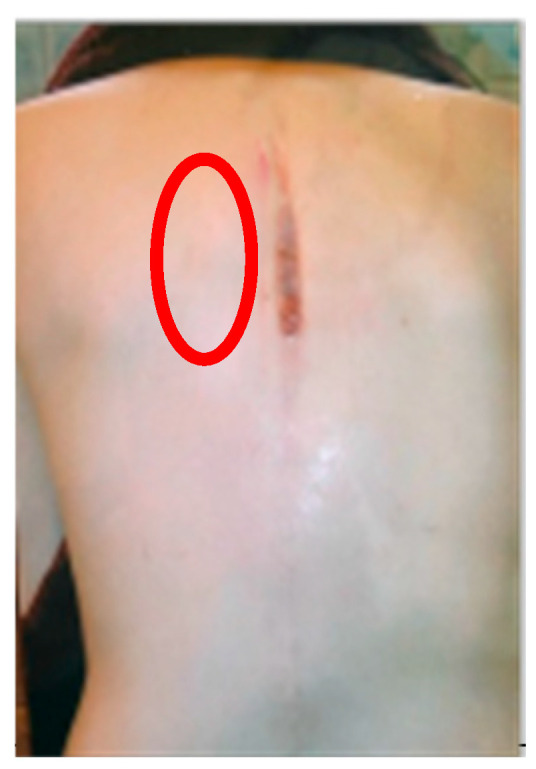
Posterior clinical photograph of the patient, showing severe infection with dehiscence of the surgical scar. The red circle highlights an area of necrosis on the left side of the spine.

Ultrasound in 2025 revealed marked unilateral atrophy of the left thoracic erector spinae at T7 (0.47 cm vs. 0.85 cm), corresponding to the structurally weakened region most affected by coronal and sagittal progression and to the site of the 2003 infection that involved wound dehiscence and soft-tissue necrosis (Figure 1).

Progressive deformity, muscular atrophy, vertical compression, and reduced trunk mobility contributed to worsening sagittal imbalance and declining quality of life. A summary of key clinical milestones, curve progression, and height changes from diagnosis through follow-up is presented in the comprehensive clinical timeline (see Table 1).

## 3. Methods

### 3.1. Patient Characteristics

The patient was a physically active, athletic 47-year-old woman with a history of AIS treated in early adulthood with posterior fusion (T5–L2) and costoplasty. A late post-operative infection required hardware removal and was followed by progressive coronal and sagittal imbalance, resulting in 80° thoracic hyperkyphosis, a 12 cm loss of height, and functional impairment. She measured 166 cm and 63.5 kg (BMI 23.0). Owing to the elevated surgical risk and prior complications, a non-operative management plan was initiated at age 47 and completed at 49.

### 3.2. Intervention Protocol (For More Details of the Protocol, Please Refer to Appendix A)

The patient completed a 28-month conservative program combining Schroth-based scoliosis-specific physiotherapy (PSSE) [22,28,43] and part-time bracing. Treatment was supervised by a Schroth-certified physiotherapist trained in four internationally recognized PSSE schools [23].

**Figure 2:**
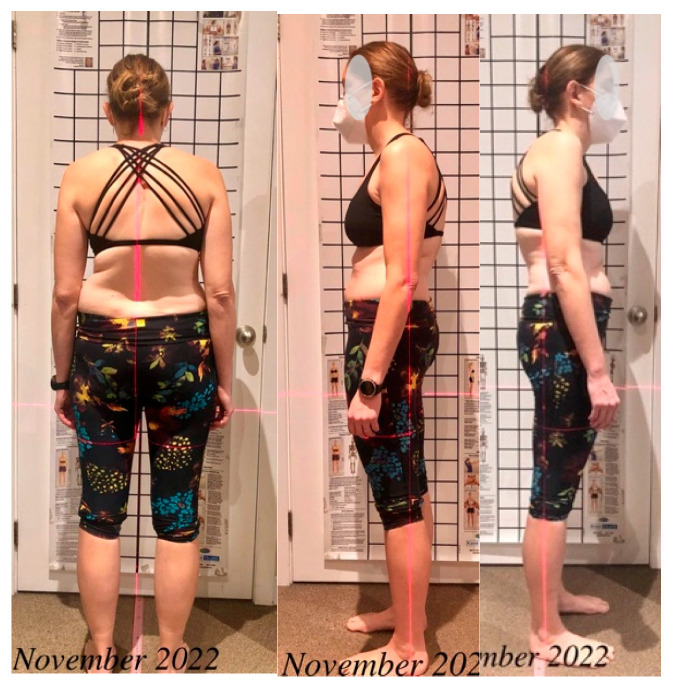
Postural photograph during initial assessment.

The physiotherapy program addressed three primary etiological factors—post-fusion segmental instability, paraspinal muscle asymmetry, and sagittal imbalance. Sagittal realignment was prioritized, as evidence consistently shows that it is the strongest determinant of disability and reduced quality of life in adult spinal deformity [44,45,46,47,48,49], thereby supporting a sagittal-focused rehabilitation strategy. The program began with a 5-day intensive phase (3 h/day), followed by a home regimen (30–60 min/day) and monthly follow-up (in person or virtual). Based on the Schroth classification [43], the patient exhibited a three-curve pattern (3C, 3cp). Exercise selection followed this classification framework and was further adapted to the patient’s specific sagittal postural deficits observed on photographic assessment. These included three-dimensional autocorrection, active self-elongation, segmental stabilization, and rotational angular breathing (RAB). Initial postural asymmetries and sagittal imbalance guiding treatment design are shown in Figure 2.

Conservative physiotherapy was first supported by a semi-rigid brace (introduced in November 2022), providing proprioceptive feedback during daily activities and sports. In January 2024, a rigid sagittal brace was added to reinforce postural correction. In February 2025, the patient was fitted with a part-time Lyon ARTBrace [34,36], a three-dimensional asymmetrical orthosis with enhanced sagittal support, intended to consolidate structural gains and maintain coronal and sagittal alignment during perimenopause, a period associated with accelerated spinal deterioration [50,51,52].

**Figure 3:**
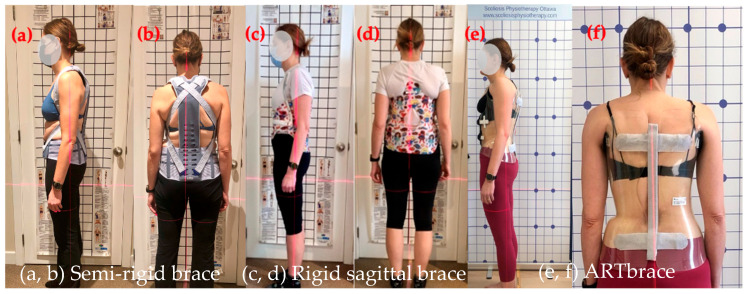
Sequential images of the patient standing in three brace types, showing the evolution of the brace design and postural response: (**a**,**b**) semi-rigid brace (November 2022), (**c**,**d**) rigid sagittal brace (January 2024), (**e**,**f**) ARTbrace (April 2025); lateral and posterior views for each.

At the end of the study period, the ARTBrace had been recently delivered, and brace-integrated Schroth exercises had not yet begun. Unlike the two previous braces, which were removed during exercise, the ARTBrace was intended to be worn for about 50% of each Schroth session to enhance postural integration and proprioceptive reinforcement. Figure 3 illustrates the three orthotic devices used. Additional details regarding brace models, objectives, and wear time are provided in Appendix A.3. These outcomes align with the emerging literature supporting bracing in adults with spinal deformities [32,34,35,36,37,53,54,55,56,57].

### 3.3. Outcome Measures

Following the SOSORT recommendations [18,23,58], a multimodal monitoring framework was implemented to evaluate structural, functional, respiratory, and quality-of-life outcomes.

Structural outcomes included radiographic Cobb angles and spinopelvic parameters (PI, PT, SS, LL, PI–LL mismatch). To reduce the variability associated with landmark ambiguity, all Cobb angles were independently measured by three evaluators. Substantial discrepancies emerged for sagittal parameters (thoracic kyphosis and lumbar lordosis). Cobb angles were used primarily for coronal curve assessment, while digital inclinometry served as the main tool to monitor sagittal alignment.

Additional structural measures included the standing height, targeted muscle ultrasound of the T7 erector spinae (2025) to assess asymmetry, trunk rotation measured with a scoliometer, and frontal balance assessed using C7 plumb-line deviation.

Respiratory function was quantified via spirometry (FVC, FEV_1_) following ERS/ATS standards.

Patient-reported outcomes comprised the Oswestry Disability Index (ODI), SRS-22 questionnaire, Trunk Appearance Perception Scale (TAPS), and Numerical Rating Scale (NRS). Technical procedures and reliability indices for all instruments are detailed in Appendix A.5 and Table A1.

#### Visual and Clinical Documentation

Although the intervention occurred within standard clinical care rather than a planned research protocol, the magnitude of change justified a retrospective, structured analysis. Longitudinal photographs and postural assessments were used to document functional and structural evolution across key timepoints.

Figures, tables, and appendices provide an integrated overview of radiological progression, postural changes, brace evolution, and functional outcomes (Figure 2, Figure 3, Figure 4, Figure 5, Figure 6, Figure 7, Figure 8 and Figure 9). Corresponding protocols and measurement procedures are detailed in Appendix A.

### 3.4. Instrument Validity and Reliability

All measurement tools were validated for adult scoliosis and spinal deformity populations, with reliability indices summarized in Appendix A.5.

Radiographs remain the gold standard for structural assessment, but their reliability is reduced in post-operative adults with severe deformity [59,60]. In this case, marked thoracic hyperkyphosis and vertebral rotation frequently obscured key landmarks (C7, T1, sacral endplate), resulting in substantial interobserver variability—especially for sagittal parameters. Additional inconsistencies arose from non-standardized imaging protocols across institutions, particularly arm positioning, which is known to influence sagittal alignment measurements [61].

Given these limitations, sagittal assessment relied primarily on digital inclinometry, which demonstrates strong inter-rater reliability, posture sensitivity, and a good correlation with radiographic values [62,63,64]. Inclinometry was paired with standardized photographic documentation to ensure consistent and meaningful longitudinal tracking throughout the intervention.

## 4. Results and Analysis

At the start of the intervention (November 2022), the patient sought to improve her sagittal and coronal balance, relieve thoracic compression (“ribs touching pelvis”), reduce exertional dyspnea, and avoid a third high-risk spinal surgery. Sagittal realignment was established as the primary therapeutic objective.

The following results summarize the structural, functional, respiratory, and quality-of-life changes observed over the 28-month conservative program combining Schroth-based physiotherapy and bracing.

### 4.1. Structural Realignment and Sagittal Correction

At initiation, clinical assessment showed advanced postural collapse and severe sagittal deformity, as confirmed by inclinometry.

After 28 months, thoracic kyphosis had decreased from 80° to 45°, with occasional post-exercise measurements reaching 41°. Lumbar lordosis increased from 47° to 53°. At baseline, PI–LL mismatch measured 15°. At follow-up, PI was 58° and LL 53°, indicating resolution of the mismatch and alignment within the SRS–Schwab target range.

Using the Global Alignment and Proportion (GAP) score [65,66,67,68], the sagittal alignment category changed from “severe” to “moderate”, with a 12° reduction in global tilt (GT). According to Schwab’s Adult Spinal Deformity Classification system [69], the number of positive sagittal modifiers decreased from three to two.

Standing height increased from 166 cm at baseline to a stable range of 169–170 cm at follow-up.

**Figure 4:**
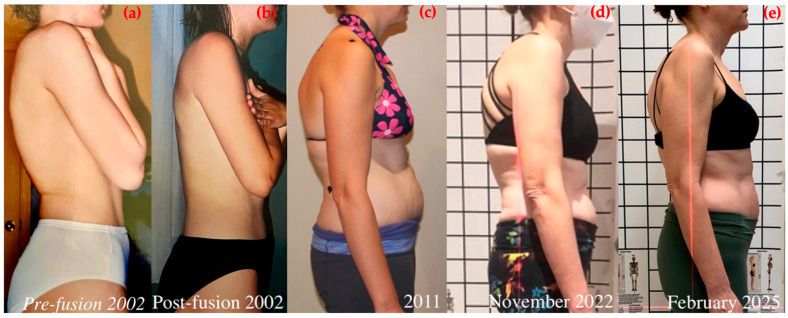
Clinical progression of kyphosis over time: posture on the thoracic convex side with the variation of the rib hump. (**a**) Pre-surgery, 2002; (**b**) post-fusion and costoplasty, 2002; (**c**) progression phase, 2011; (**d**) pre-Schroth and pre-bracing, November 2022; (**e**) after 28 months of conservative treatment, February 2025.

**Figure 5:**
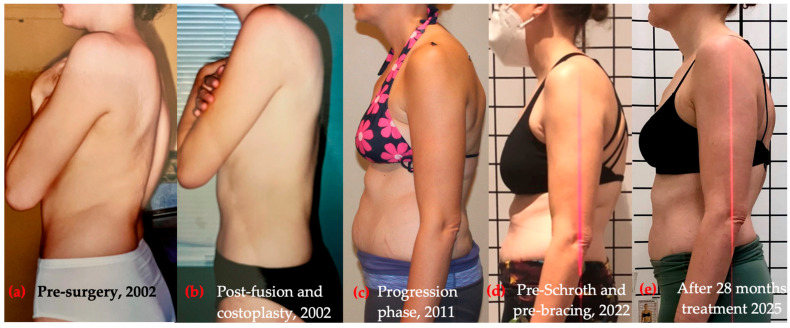
Clinical progression of kyphosis over time with photo of posture on thoracic concave side. (**a**) Pre-surgery, 2002; (**b**) post-fusion and costoplasty, 2002; (**c**) progression phase, 2011; (**d**) pre-Schroth and pre-bracing, November 2022; (**e**) after 28 months of conservative treatment, February 2025.

Standardized lateral photographs provided visual documentation of sagittal postural correction, complementing quantitative alignment measures (from year to year). Representative images illustrating kyphosis and rib hump changes from convex and concave perspectives are shown in Figure 4 and Figure 5.

### 4.2. Coronal Alignment and Rib Cage Remodeling

Although sagittal realignment was the primary therapeutic target, coronal parameters also changed over time. The thoracic curve decreased from 48° (2016) to 32° Cobb (2025), representing a 16° reduction, while the lumbar curve remained stable at approximately 34° Cobb. Coronal imbalance resolved, with the C7 plumb line shifting from 3.5 cm left of the midline to 0 cm.

Rib cage geometry was assessed and also demonstrated structural remodeling using Mehta’s Rib–Vertebra Angle Difference (RVAD) method [70] (typically applied in pediatrics) at the T10 apex. The concave rib angle improved from 80° to 90° and the convex rib from 65° to 80°, reducing the RVAD from 15° to 10°.

**Figure 6:**
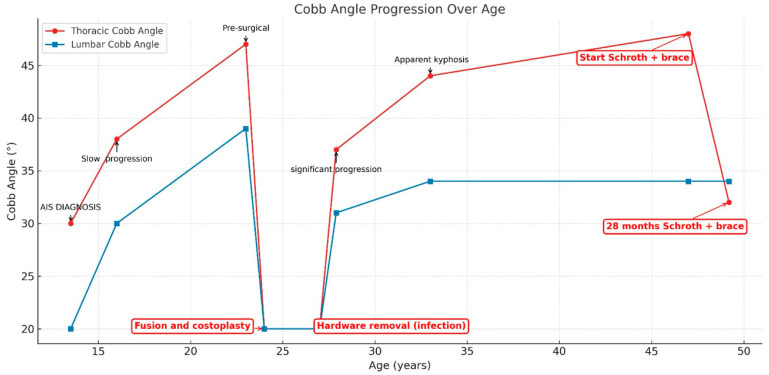
Lumbar and thoracic Cobb angles changes on radiographs taken between 1988 and 2025 (13–49 years).

These changes are summarized in Figure 6, which illustrates the thoracic and lumbar Cobb angles across radiographs obtained between 1988 and 2025 (ages 13–49). Trunk asymmetry, assessed with a scoliometer, remained stable at approximately 15° ATR. Due to prior costoplasty, ATR values were considered approximate and used primarily for trend monitoring.

### 4.3. Functional, Respiratory, and Neurological Outcomes

By the end of the intervention, the patient reported full physical capacity, including running, swimming, and cycling, without imbalance or exertional dyspnea. Symptoms of gastroesophageal reflux also diminished during the intervention period.

Vital capacity increased from 2700 mL to 3600 mL (+33%), with chest expansion improving by approximately one third, in line with previous reports on respiratory change in adults treated with Schroth-based physiotherapy [18,22,71].

Neurologically, mild L5–S1 motor weakness and bilateral hyperreflexia remained present but stable over time, without evidence of upper motor neuron involvement.

### 4.4. Quality of Life and Functional Recovery

The Oswestry Disability Index (ODI) scores reflected longitudinal disability changes. In 2003, at the pre-surgical peak, disability reached 66%, associated with severe pain related to infection and osteomyelitis requiring hardware removal. The ODI improved to 0% within one year post-operatively and was then measured at 4% in late 2022, immediately before starting Schroth-based therapy. After 28 months of conservative treatment, the ODI returned to 0% and remained at this level through 2025.

SRS-22 scores improved across all domains between baseline and final follow-up: pain (2.5 → 4.5), function (2.8 → 4.2), self-image (2.3 → 4.4), mental health (4.4 → 5.0), while satisfaction remained unchanged (5.0).

### 4.5. Integrated Timeline and Multimodal Monitoring

A visual timeline (Table 1, in the case presentation Section 2.5) summarizes clinical milestones from post-hardware collapse to the end of conservative management. It aligns key treatment phases—including brace changes and physiotherapy progression—with corresponding structural and functional measures.

**Figure 7:**
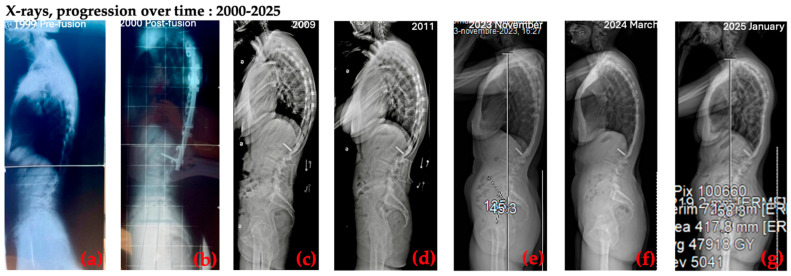
Sagittal X-ray progression over time: (**a**) pre-surgery, 1999; (**b**) post-surgery, 2000; (**c**,**d**) sagittal postural deterioration, 2009 and 2011; (**e**) sagittal postural improvement, after 1 year Schroth + brace; (**f**) sagittal postural improvement, after 17 months Schroth + brace; (**g**) sagittal postural improvement, after 28 months Schroth + brace.

**Figure 8:**
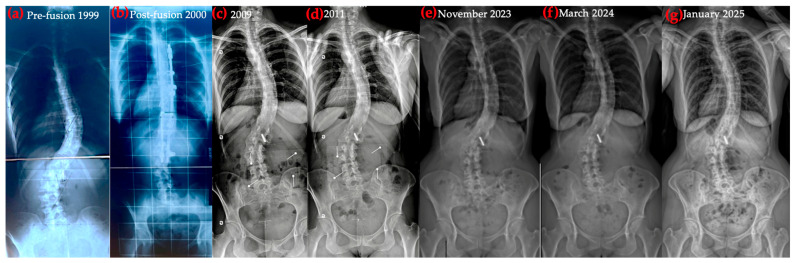
Coronal X-ray progression over time: (**a**) pre-surgery; (**b**) post-surgery; (**c**,**d**) coronal postural deterioration; (**e**) coronal postural improvement, after 1 year Schroth + brace; (**f**) coronal postural improvement, after 17 months Schroth + brace; (**g**) coronal postural improvement, after 28 months Schroth + brace.

**Figure 9:**
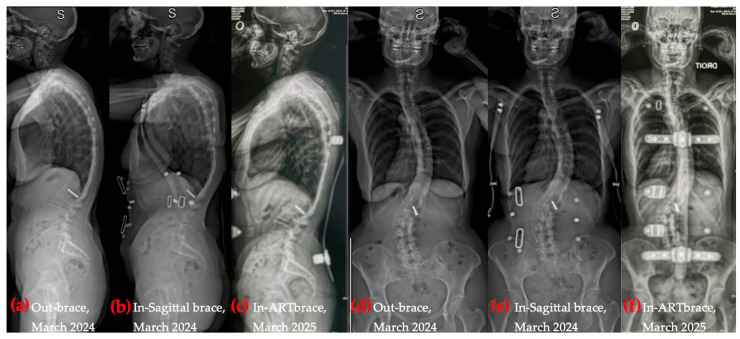
Coronal and sagittal radiographic progression over time, with and without bracing. (**a**) Out-of-brace, sagittal X-ray; (**b**) In sagittal brace, sagittal X-ray; (**c**) In ARTbrace, sagittal X-ray; (**d**) Out-of-brace, coronal X-ray; (**e**) In sagittal brace, coronal X-ray; (**f**) In ARTbrace, coronal X-ray.

Longitudinal coronal and sagittal outcomes were documented using standardized radiographs and clinical photographs at five intervals: pre-operative, post-fusion with costoplasty, progressive deformity phase, pre-intervention, and post-treatment review. As shown in Figure 4, Figure 5, Figure 6, Figure 7, Figure 8 and Figure 9, outcomes ranged from early post-fusion postural improvement to deterioration after hardware removal, followed by measurable realignment achieved through Schroth-based physiotherapy and bracing between 2000 and 2025.

### 4.6. Summary of Clinical Outcomes

Across structural, respiratory, and functional domains, the patient achieved substantial recovery. Thoracic kyphosis decreased from 80° to 45° (−43.8%) and the thoracic curve from 48° to 32° (−33%), and vital capacity increased by 900 mL (+33%). The ODI improved from 4% to 0%, confirming complete functional recovery maintained for more than two years. SRS-22 scores improved across all domains—pain, function, self-image, and mental health—reflecting consistent quality-of-life gains.

Together, these outcomes demonstrate that Schroth-based physiotherapy combined with individualized part-time bracing can produce meaningful structural correction and functional restoration in complex adult post-fusion scoliosis after hardware removal.

## 5. Discussion

### 5.1. Novelty and Key Contributions

This case describes a post-fusion adult scoliosis patient who developed rapid sagittal collapse and hyperkyphosis after hardware removal and who subsequently achieved meaningful structural and functional improvements through a structured conservative program. To our knowledge, this is the first documented case showing the restoration of sagittal alignment, respiratory function, and quality of life following complete implant removal in an adult scoliosis patient treated exclusively with physiotherapeutic scoliosis-specific exercises (PSSE) and individualized bracing. This report also addresses a patient subgroup not represented in the current SOSORT or SRS guidelines and illustrates the potential role of non-surgical management in selected adults after hardware removal.

### 5.2. Rationale for Choosing the Schroth Method

The Schroth-based approach was selected based on clinical reasoning and therapist expertise. Although prior conservative treatment, including Global Postural Re-education, had been attempted, the patient did not experience sufficient or sustained improvement. Following infection-related complications and subsequent hardware removal, the patient pursued a non-surgical alternative with the goal of avoiding a third operative intervention. The treating physiotherapist held certifications across four major PSSE schools [23] and, having been mentored by Christa Lehnert-Schroth [43], brought extensive clinical and academic experience within SOSORT.

Given the absence of evidence-based guidelines for adults after fusion and hardware removal, a customized strategy emphasizing three-dimensional autocorrection, sagittal realignment, and respiratory training was warranted to address postural collapse and mechanical imbalance.

### 5.3. Mechanisms of Improvement

Sagittal realignment was prioritized because sagittal imbalance is more strongly associated with pain, disability, and reduced quality of life than coronal deformity in adults with spinal deformities. The combination of 3D autocorrection, rotational angular breathing (RAB), and part-time bracing likely improved lumbar extension function, reduced compensatory trunk flexion, improved diaphragmatic excursion and chest mobility, decreased vertical collapse forces, and improved trunk control, collectively explaining the observed structural and functional recovery.

### 5.4. Relation to Existing Literature

This case illustrates the potential for major structural deterioration following hardware removal, especially without timely re-instrumentation. In this patient, the thoracic and lumbar curves progressed by 17° and 11° within nine months, far exceeding the long-term losses typically reported after Harrington instrumentation: 7° over 20 years (Helenius et al. [72]), 5–10° over 20–30 years (Mariconda et al. [73]), and 9.5° on average (Barile et al. [74]). Infection likely accelerated deterioration, consistent with Muschik et al. [3], who observed worsening deformity in 82% of late infections despite re-instrumentation, and Alpert et al. [8], who reported greater Cobb losses in infection versus non-infection cases (33.8° vs. 18.8°).

Over 13 years, the thoracic and lumbar curves progressed by 28° and 14°, returning to near-pre-operative magnitudes. The most pronounced degeneration occurred between T2 and T8, corresponding to the previously necrotic zone (Figure 1). Muscle ultrasound in 2025 revealed localized paraspinal atrophy at T7 (0.47 cm vs. 0.85 cm), consistent with concave-side weakness, and this may have contributed to mechanical instability. This pattern aligns with findings from Cheung et al. [75], whose preliminary EMG study reported greater erector spinae activation on the convex side—particularly at the upper-end vertebra, apex, and lower-end vertebra—an asymmetry associated with curve progression.

Despite this unfavorable context, the patient achieved substantial realignment under conservative care. These gains compare favorably with surgical series reporting sagittal corrections of 30°–50° but accompanied by significant risks, such as proximal junctional failure, pseudarthrosis, and other post-operative complications [76,77]. In contrast, this patient achieved comparable alignment gains non-invasively and without adverse events.

### 5.5. Case-Specific Factors and Limitations

As a single-case report, generalizability is limited. Radiographic assessment was affected by anatomic distortion, vertebral rotation, costoplasty, and inconsistent positioning, all of which increased variability; ATR was monitored but used for trends only. Exercise and brace adherence were self-reported, and the patient’s dual role (patient and lead author) introduces potential bias. The patient’s residence approximately 525 km from the clinic, limited the feasibility of standardized follow-up and imaging. The treatment plan evolved clinically rather than being pre-defined for research, and early imaging is incomplete. Observation ended prior to ARTBrace initiation to avoid confounding. Care occurred without specific guidelines for adults with post-fusion scoliosis or hardware removal.

### 5.6. Confounding Factors and Additional Considerations

Generalized joint hypermobility [78] (Beighton score: 6/9) likely affected both postural decline and subsequent recovery. Its impact on proprioception and spinal stability may heighten deformity risk yet enhance responsiveness to targeted physiotherapy, emphasizing the value of individualized rehabilitation in complex adult spinal deformity. 

This case also supports emerging evidence that pelvic incidence (PI) may not be a fixed anatomical constant. In this patient, PI decreased from 62° to 58° over the treatment period, consistent with the concept of spinopelvic plasticity described in the recent literature. Manzetti et al. (2025) [79] reported PI changes greater than 5° in nearly half of adults with spinal deformity, suggesting adaptive sacropelvic adjustments under mechanical or therapeutic influences. Additionally, Lee et al. [80] demonstrated that horizontal and vertical pelvic rotations during radiographic acquisition can produce apparent PI variations of up to 6°, indicating that part of the observed change may reflect positional or methodological artifacts rather than true anatomical modification.

### 5.7. Clinical and Research Implications

Adults with post-fusion scoliosis or complete hardware removal remain largely absent from existing research frameworks and are not represented in the current clinical guidelines. This case suggests that a sagittal-focused conservative strategy—including PSSE, modern bracing, and multidisciplinary follow-up—may offer a viable alternative or complement to revision surgery in carefully selected adults. The meaningful structural, respiratory, and functional improvements observed in this patient indicate that non-surgical management may hold therapeutic potential even in complex presentations traditionally considered surgical.

Several clinical and scientific implications emerge from these findings. Future research is needed to better define which adults are most likely to benefit from conservative management after fusion or implant removal and to clarify the optimal “dose” and training parameters associated with PSSE-based interventions. Comparative work examining the effects of different brace designs on sagittal realignment and symptom reduction would further strengthen the evidence base. In addition, the evolving understanding of spinopelvic dynamics—including the possibility of pelvic incidence variability—warrants investigation through longitudinal, three-dimensional imaging to distinguish true anatomical adaptation from radiographic or positional artifacts. Ultimately, developing standardized, evidence-informed care pathways for this underserved subgroup is essential to improve access, consistency, and clinical decision-making in the non-surgical management of adult scoliosis after hardware removal.

### 5.8. Proposed Post-Fusion Adult Scoliosis Protocol

Based on this case, an individualized multimodal framework may be proposed for adults with post-fusion scoliosis, hardware removal, or revision surgery. Management should combine scoliosis-specific exercises targeting posture, sagittal alignment, core stability, and respiratory mechanics with orthotic support when indicated, supported by multidisciplinary review every 3–6 months.

This framework helps to bridge current practice gaps and may guide the development of future guidelines for complex adult scoliosis.

## 6. Conclusions

This case shows that a structured conservative management—integrating Schroth-based physiotherapy and individualized bracing—can achieve meaningful improvements in posture, respiratory function, and quality of life in complex post-fusion scoliosis after hardware removal, thereby avoiding high-risk revision surgery. These results support conservative care as a viable, lower-risk alternative for selected adults and reinforce the need for updated clinical guidelines addressing this underrepresented patient population.

## Figures and Tables

**Table 1:** Summary of clinical timeline, interventions, and patient outcomes (2002–2025). Sagittal kyphosis values are derived from inclinometer measurements rather than Cobb angles due to limited radiographic visibility (see Section 3.4 for details).

Year	Age	Clinical Phase	Thoracic Kyphosis (° Inclinometry)	Thoracic Curve (° Cobb) X-Ray	Lumbar Curve (° Cobb) X-Ray	Standing Height (cm)	Brace Type	ODI (%)	Vital Capacity (mL)
2000 (May)	24	Post-Fusion Surgery (T5–L2 + Costoplasty)		20	20	178	—	Pre-op: 2 Post-op: 6	—
2002 (July)	27	Post-Partum Infection						—	
2003 (February)	28	Osteomyelitis, Necrosis/Hardware Removal		20	20	178	—	Pre-op: 66 Post-op: 16	—
2003 (September)	28	Early Post-Removal Curve Progression		37	31	—	—	0	—
2016	41	Ongoing Structural Deterioration		48	34	—	—	—	—
2022 (November)	47	Initiation Schroth Physiotherapy + Bracing	80°	-	-	166	Semi-Rigid **	4	2700
2023	48	During Intervention: Schroth Physiotherapy + Bracing	62° *	43° (November)	36° (November)	170	Sagittal rigid ***	0	3600
2024	48	During Intervention: Schroth Physiotherapy + Bracing	61° *	41° (March)	41° (March)	170	Sagittal rigid ***	0	3300 *****
2025 (February)	49	During Intervention: Program End of 28-Month	45°	32°	34°	170	ARTbrace ****	0	3600

* Average for the year. ** Proprioceptive semi-rigid brace, November 2022. *** Sagittal rigid brace, January 2023. **** ARTbrace February 2025. ***** Whooping cough, pneumonia for several months.

**Figure A1:**
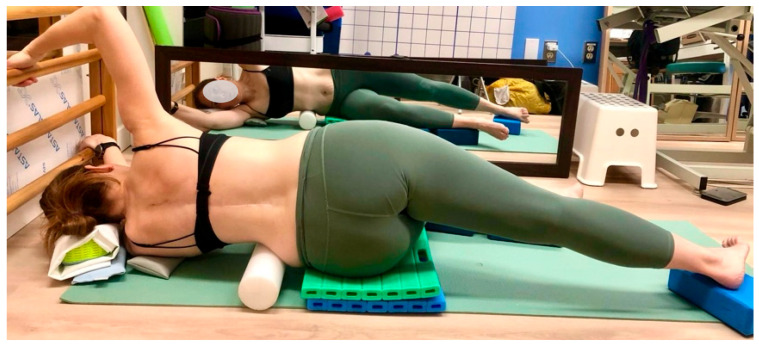
Side-lying Schroth exercise for major thoracic and minor lumbar scoliosis.

**Figure A2:**
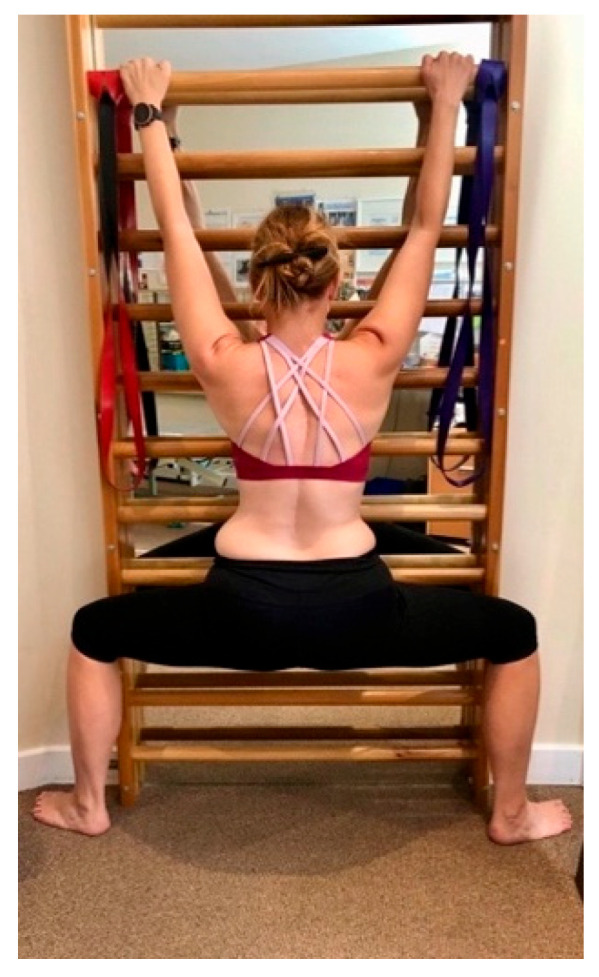
Modified semi-hanging-Schroth method.

**Figure A3:**
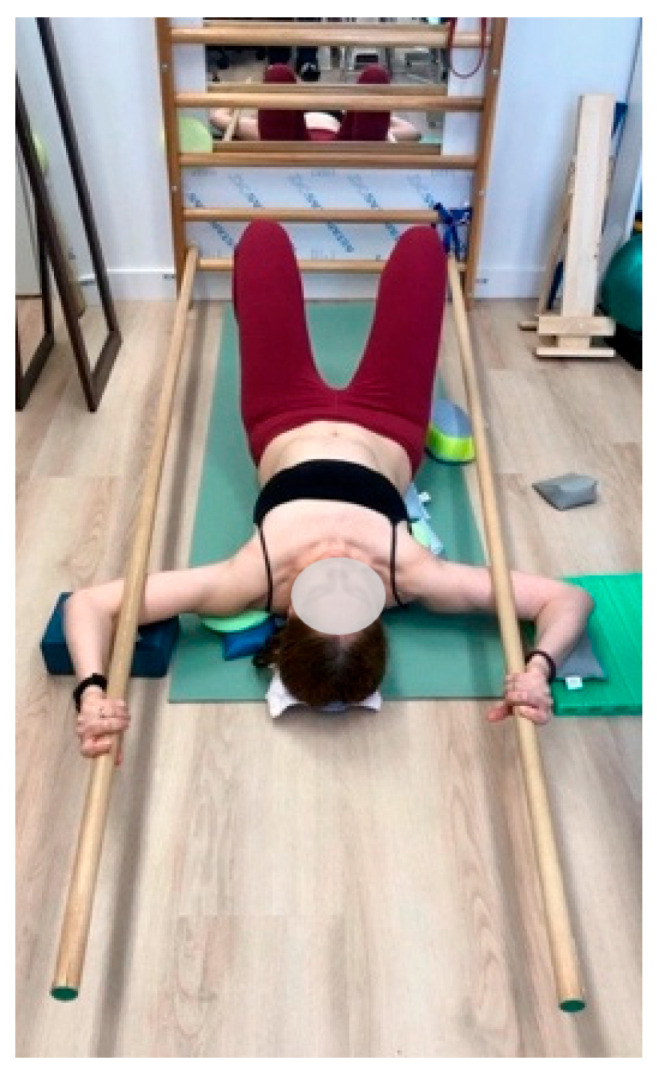
Supine with two poles with bilateral shoulder traction for hyperkyphosis and right thoracic–left lumbar scoliosis—Schroth method.

**Figure A4:**
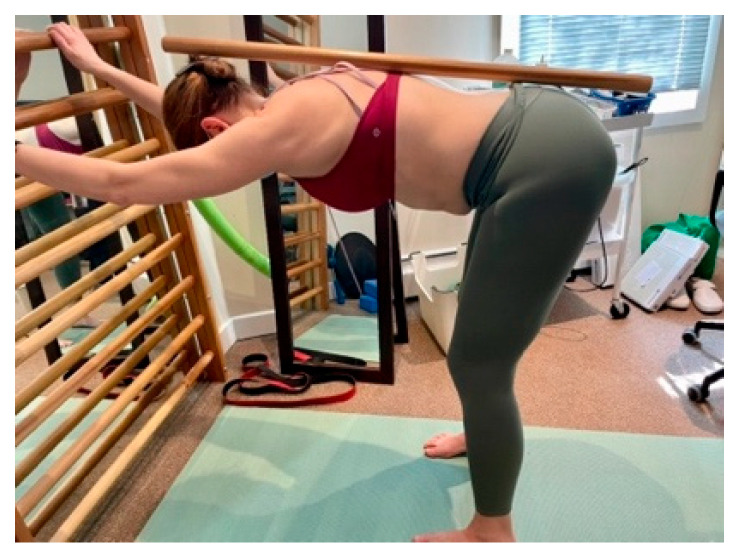
Prone on feet—Schroth and Lyon method exercise.

**Figure A5:**
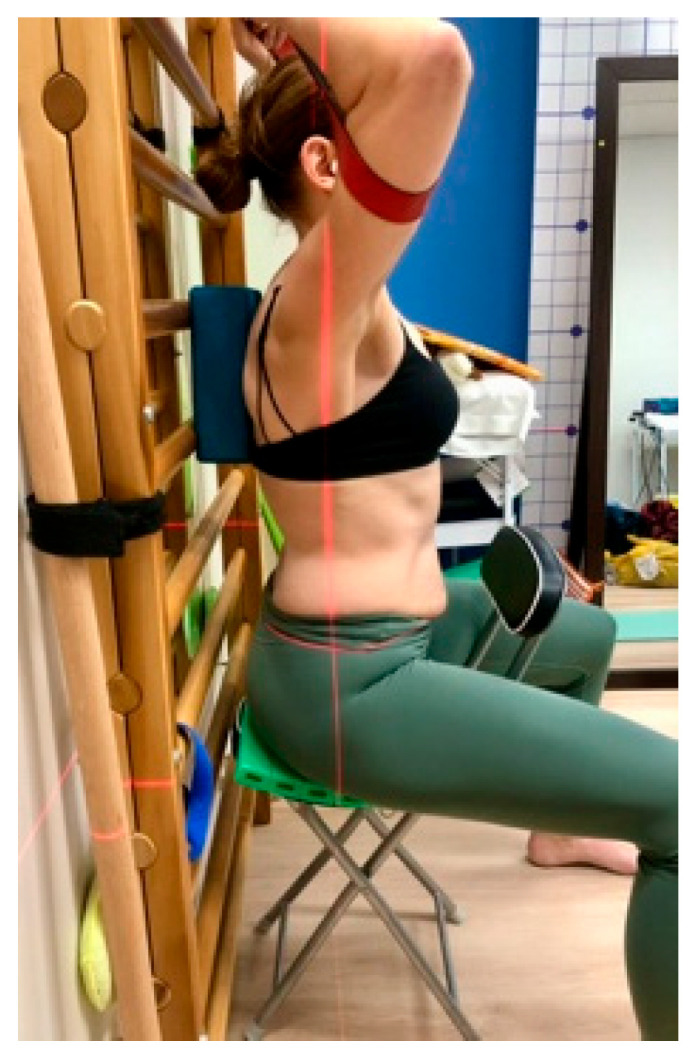
Reverse sitting with thoracic apex support and elastic resistance—Schroth method exercise for hyperkyphosis.

**Figure A6:**
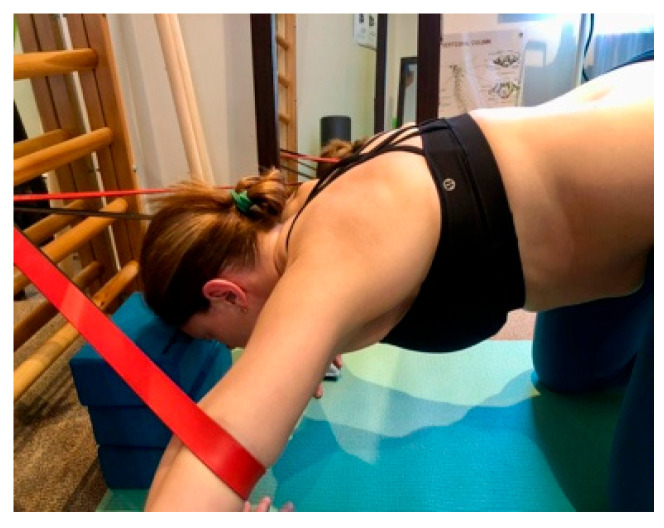
Iguana position in quadruped—Schroth method.

**Figure A7:**
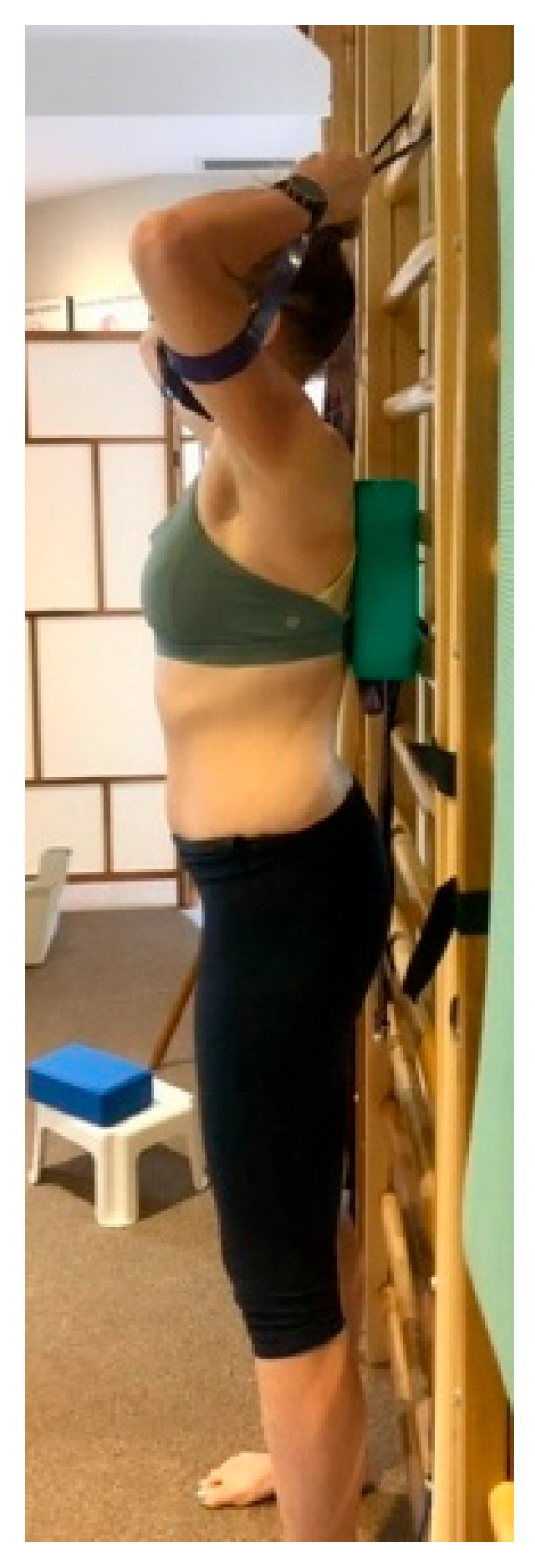
Reverse standing with thoracic apex support and elastic resistance—Schroth method exercise for hyperkyphosis.

**Figure A8:**
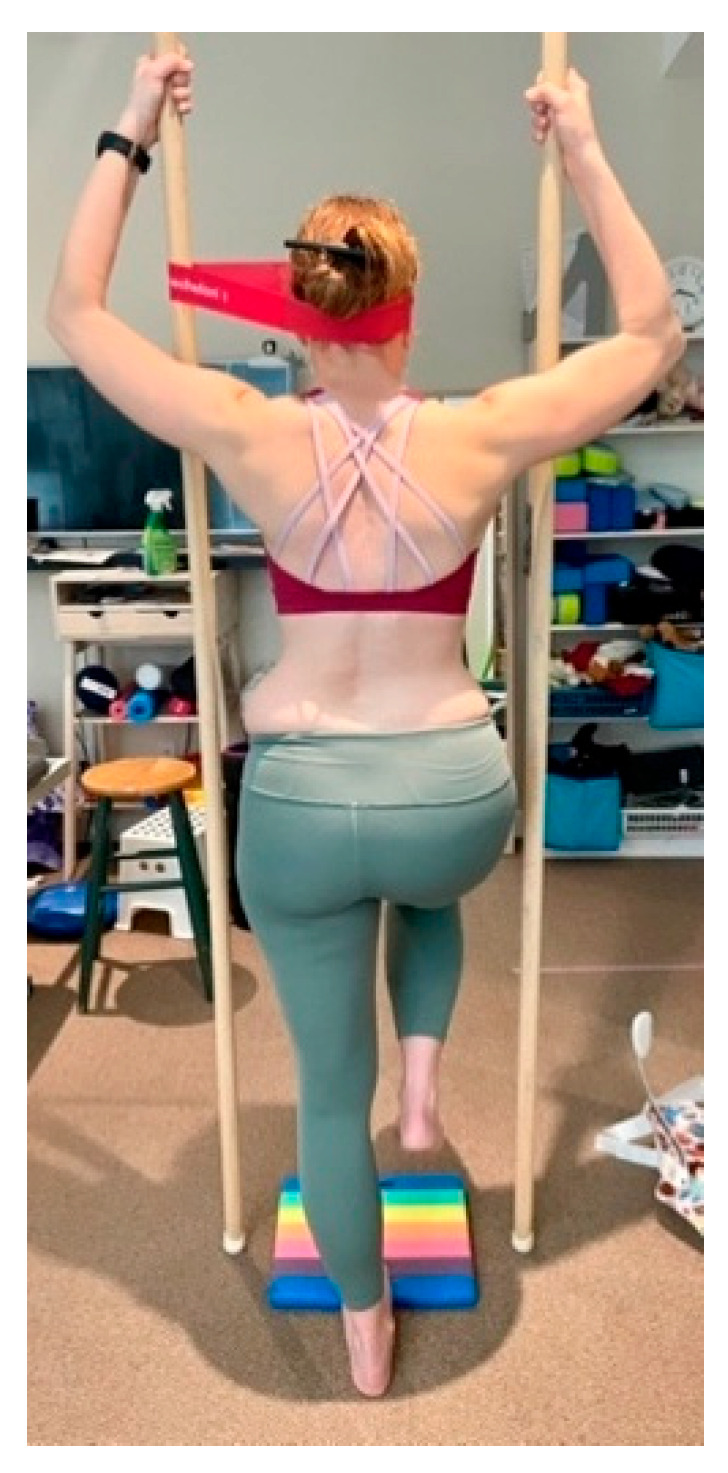
Standing on one foot—Schroth exercises.

## Data Availability

All data supporting the findings of this case study are included within the article. Additional de-identified clinical data may be available from the corresponding author upon reasonable request.

## Appendix A. Schroth-Based Intervention Protocol

The conservative treatment program followed by the patient included a combination of scoliosis-specific physiotherapy (Schroth method) and bracing over a 28-month period (November 2022 to February 2025). The intervention was structured as follows.

### Appendix A.1. Initial Phase

- Duration: 5 consecutive days
- Format: 3 h per day of supervised therapy
- Provider: Certified Schroth physiotherapist
- Objectives: Postural education, sagittal realignment, breathing mechanics, patient-specific exercise prescription

### Appendix A.2. Ongoing Home Program

- Daily home exercises: 30 to 60 min per day
- Follow-up sessions: Monthly (in-person or virtual) with the Schroth therapist
- Compliance: Self-reported, with high adherence noted throughout the program

### Appendix A.3. Bracing Strategy

- Semi-rigid brace:
  ○ Used part-time (6–12 h/day) from November 2022
  ○ Aimed at proprioceptive feedback during ADLs and sports
  ○ Not worn during Schroth exercises
- Rigid sagittal brace:
  ○ Introduced in January 2024
  ○ Worn 10–15 h/day
  ○ Continued semi-rigid brace use for sports activities
  ○ Targeted sagittal plane restoration
  ○ Not worn during Schroth exercises
- Rigid sagittal brace (ARTbrace):
  ○ Introduced in February 2025
  ○ Worn for 24 h/day minus 10 min during the first month, then 4 to 6 h a day and always 2 consecutive hours after practicing sports without the brace
  ○ Worn for half of the Schroth PSSE
  ○ Targeted three-dimensional correction

### Appendix A.4. Selected Schroth Exercises with Descriptions

The following section presents a selection of Schroth-based scoliosis-specific exercises (PSSE) that were prescribed and regularly performed by the patient during the 28-month intervention period. Each exercise is illustrated with a photograph and accompanied by a brief description outlining its therapeutic objective. The exercises presented here are a representative selection of those performed by the patient; they do not encompass the full program followed throughout the intervention. However, this subset reflects the key therapeutic elements that, in combination with part-time bracing, contributed to the structural and functional improvements documented in this case.

Rooted in three-dimensional postural correction, the Schroth method emphasizes spinal elongation, rotational angular breathing (RAB), and asymmetrical muscle activation to address scoliosis-specific imbalances. Exercises are tailored to the individual’s curve pattern and postural presentation, aiming to improve trunk alignment, muscular symmetry, and postural awareness in both static and dynamic contexts. (Figure A1, Figure A2, Figure A3, Figure A4, Figure A5, Figure A6, Figure A7 and Figure A8).

Each Schroth exercise session begins with a 10 min warm-up, followed by coordination-based exercises, Schroth-specific directional breathing, muscle activation, and semi-hanging.

Figure A1 illustrates the side-lying exercise described below.

Objectives

- Open the thoracic and lumbar concavities through positioning and directional breathing.
- Create isometric muscle tension around convexities to centralize the thorax and spine.
- Lengthen shortened hip abductors and activate the concave of the lumbar curve quadratus lumborum (QL).
- Promote three-dimensional structural correction and neuromuscular re-education.

Position

- Patient lies on the thoracic concave side.
- Support pads are placed under the lumbar convexity, main thoracic, and upper thoracic regions to limit compensatory curvature.
- The right leg is positioned on a yoga block, which helps to lengthen shortened hip abductors.

Actions

- Perform Schroth-specific directional breathing into the concavities.
- Engage isometric contraction around the convexities to bring the spine closer to the midline.
- When the right leg is lifted, activate the concave lumbar concavity quadratus lumborum (QL).
- Maintain corrective alignment throughout the exercise.

Figure A2 illustrates the modified semi-hanging exercise described below.

Objective

- Axial elongation, pelvic centering.

Position

- Patient with abducted hips, pelvis centered.

Actions

- Perform elongation during exhalation.

Precautions

- Avoid in patients with spinal fusion unless clinically indicated.

Figure A3 illustrates supine with two poles exercise described below.

Objective

- Reduce hyperkyphosis and optimize three-dimensional scoliosis correction through biomechanical forces and Schroth-specific breathing.

Position

- Supine with a stable, neutral pelvis and padding to maintain lumbar lordosis.
- Axial elongation (cranial traction) and caudal traction (toward the feet).
- Arms are positioned for left thoracic shoulder traction and right thoracic shoulder counter-traction with physiologically stable scapulae, supporting axial elongation and thoracic expansion; this arm position and muscle activation contribute to self-elongation and help to prevent postural collapse.

Actions

- Perform Schroth breathing with focus on expanding concavities.
- Apply bilateral shoulder muscle activation to enhance axial elongation and lateral thoracic expansion.
- Engage muscle activations around convexities to guide curves inward and forward, preventing postural collapse and promoting more neutral spinal re-alignment.

Figure A4 illustrates prone on feet exercise described below.

Objective

- Sagittal posture control, spinal awareness with trunk flexion.
- Position
- Patient bends trunk forward 90° with pole along spine (head, thoracic apex, sacrum contact).

Actions

- Extend knees as much as possible while maintaining sagittal profile and maintaining pole contact with the head, the thoracic apex, and the center of the sacrum.

Figure A5 illustrates reverse sitting exercise described below.

Objective

- Reduce thoracic hyperkyphosis, strengthen postural control.

Position

- Patient seated in reverse with yoga block at thoracic apex of the thoracic kyphosis to provide firm pressure toward correction; elastic band pulled downward to assist axial elongation.

Actions

- At rest, the patient inhales and slowly exhales during muscle activation.

Figure A6 illustrates Iguana exercise described below.

Objective

- Enhance spinal elongation, shoulder stabilization, strengthening.

Position

- Patient is in a quadruped (hands and knees) position with a resistance band to assist spinal elongation, shoulder stabilization, and strengthening.

Actions

- Perform axial elongation and controlled breathing.

Figure A7 illustrates reverse standing (a variation of reverse sitting), demonstrating the full postural control exercise described below.

Objective

- Improve sagittal alignment, strengthen axial elongation.

Position

- Patient stands against a yoga block at the thoracic apex of the thoracic kyphosis to provide firm pressure toward correction, with elastic band pulled downward.

Actions

- Maintain sagittal alignment during Schroth breathing while using an elastic resistance band to assist axial elongation through downward traction, ensuring full postural control with elastic support. At rest, the patient inhales, while they slowly exhale during muscle activation.

Figure A8 illustrates Standing on one foot exercise described below.

Objective

- Improve sagittal alignment, postural correction, and a centered head position.

Position

- Patient stands on one foot with poles, with pad under forefoot to shift weight toward the heel (which facilitates improved sagittal alignment). An elastic band around the head helps to maintain a midline head position.

Actions

- Shift weight to heel and press poles downward, supporting axial elongation and full postural correction; alternate legs while maintaining full postural correction.

### Appendix A.5. Measurement and Monitoring Tools

To monitor outcomes throughout the intervention, a combination of objective, functional, and patient-reported measures was employed. All instruments were selected based on their clinical relevance and documented validity in adult spinal deformity populations. Calibration intervals and reliability indices are summarized in Table A1.

Structural Measures

Digital Inclinometry: Thoracic kyphosis and lumbar lordosis angles were measured in relaxed and corrected standing positions with the patient barefoot and arms resting naturally along the body using a digital inclinometer (Baseline Digital Inclinometer).

- Thoracic kyphosis was measured by placing the inclinometer at the spinous processes of T1–T2 (superior landmark) and T12–L1 (inferior landmark) and calculating the angular sum.
- Lumbar lordosis was measured from T12–L1 (superior landmark) to S1 (inferior landmark), using the same angular sum method.

Readings were performed by the same physiotherapist to minimize inter-rater variability. Measurements were taken three times and averaged. This method shows good reproducibility in adults, with reported intra-rater ICC of 0.88 and inter-rater ICC of 0.86.

- Frontal Imbalance: Manual ruler method assessing C7 plumb-line deviation from the sacrum in frontal view.
- Scoliometer (Angle of Trunk Rotation): Used to assess trunk rotation. ICC values for scoliometer use in adult scoliosis populations exceed 0.85.
- Standing Height: Measured barefoot using a stadiometer (Hopkins Medical). Tracked longitudinally and annotated in the clinical timeline.
- Photographic Documentation: Frontal and sagittal posture images were taken at every in-person session, both before and after each Schroth physiotherapy session, using an iPhone 10. All photographs were captured under standardized conditions: barefoot, in a neutral stance, with the camera positioned 2 m from the patient. This protocol was implemented to consistently document postural evolution and to assess the immediate and cumulative effects of Schroth exercises over time. Representative changes are illustrated in Figure 4 and Figure 5.
- Ultrasound Imaging: Used to assess muscle thickness (erector spinae and psoas major) as a proxy for asymmetry and functional recovery. Scans performed by the same person (GE Venue Go R3 probes C1-5 and ML 6-15).
- Radiographs were used to quantify structural parameters including Cobb angles and spinopelvic metrics (pelvic incidence, pelvic tilt, sacral slope, lumbar lordosis, and PI–LL mismatch). Standardized full-spine protocols were followed.

Respiratory Measures

- Spirometry: Forced vital capacity (FVC) and forced expiratory volume in one second (FEV_1_) were recorded using a calibrated Sammons Preston Buhl spirometer. Testing followed European Respiratory Society/American Thoracic Society guidelines for validity and reproducibility. Test–retest ICC for FVC was 0.94; inter-rater ICC was 0.92.

Patient-Reported Outcomes

- SRS-22 Questionnaire: Evaluated function, pain, self-image, mental health, and satisfaction with treatment. Demonstrates high reliability in adult populations (ICC: 0.87 intra-rater; 0.85 inter-rater).
- Oswestry Disability Index (ODI): Used to measure functional impairment. Validated in chronic low back pain and spinal deformity cohorts (ICC: 0.92 intra-rater; 0.91 inter-rater).
- TAPS (Trunk Appearance Perception Scale): Captured subjective perception of trunk asymmetry.
- Numerical Rating Scale (NRS): Monitored pain intensity during functional activities.

All instruments were used according to manufacturer specifications and calibrated at regular intervals to ensure measurement accuracy and reproducibility.

**Table A1:** Summary of measurement tools and reliability.

Measurement Tool	Model/Manufacturer	Calibration Interval	ICC (Intra-Rater)	ICC (Inter-Rater)
**Digital Inclinometer**	Baseline Digital Inclinometer	Annually	0.88	0.86
**Manual Ruler (C7 Plumbline)**	Standard	Not applicable	0.87	0.85
**Scoliometer (ATR)**	Bunnell	Annually	0.85	0.86
**Portable Spirometer**	Sammons Preston Buhl	Quarterly	0.94	0.92
**Stadiometer**	Hopkins Medical	Annually	N/A	N/A
**Ultrasound Imaging**	GE Venue Go R3 probes C1-5 and ML 6-15	Bi-annually	0.91	0.88
**iPhone 10 (Photographs)**	Apple	Not applicable	N/A	N/A
**SRS-22 Questionnaire**	N/A	Not applicable	0.87	0.85
**Oswestry Disability Index (ODI)**	N/A	Not applicable	0.92	0.91
**TAPS**	N/A	Not applicable	0.88	0.84
**Numerical Rating Scale (Pain)**	N/A	Not applicable	0.89	0.87

### Appendix A.6. Therapeutic Goals

- Sagittal realignment
- Frontal plane balance
- Reduction in thoracic–pelvic contact
- Improved respiratory function
- Maintenance of postural alignment and previously achieved corrections during menopause and age-related high-risk transition periods
- Avoidance of third invasive surgery
